# Reading to Translate or Translating to Read? Modeling Translators’ Eye Movements with Multilingual Pre-Trained Models

**DOI:** 10.3390/jemr19030066

**Published:** 2026-06-08

**Authors:** Yiyu Zhang, Xiajing Yao, Dechao Li

**Affiliations:** 1 School of Foreign Languages, China University of Geosciences (Wuhan), Wuhan 430074, China; yiyu.zhang@polyu.edu.hk (Y.Z.); yaoxiajing@cug.edu.cn (X.Y.); 2 Department of Language Science and Technology, The Hong Kong Polytechnic University, Hong Kong SAR, China

**Keywords:** eye-tracking, surprisal, computational psycholinguistics, bilingual reading, translation process, post-editing, language model, neural machine translation

## Abstract

Translation and post-editing both integrate reading into bilingual text production, yet it remains unclear which computational predictors from multilingual pre-trained models best account for translators’ reading patterns across task types and translation directions. We recruited twenty-six Chinese L1 translators who completed en→zh and zh→en translation and post-editing tasks, yielding 104 eye-tracking sessions. Dependent measures were source reading time (TrtS), target reading time (TrtT), and target production duration (Dur). Predictors were derived from two model architectures, a decoder-only language model (LM) and an encoder–decoder neural machine translation (NMT) model, and they included monolingual surprisal, translation surprisal with source context, and attention features computed from models’ internal weights. Analyses showed that LM surprisal provided the strongest account of target reading, while source reading was most strongly predicted by encoder self-attention with LM surprisal, a robust secondary predictor, and target production duration drew on both LM and NMT translation surprisal. Direction effects were broader than task effects, especially on target measures. These findings suggest that although translation reading is bilingual in task structure, cumulative reading time is best explained by monolingual LM surprisal, whereas production duration additionally reflects NMT translation surprisal and revision behavior.

## 1. Introduction

Eye movements during reading reveal the cognitive processes that support language comprehension [1,2]. Research has examined word recognition and sentence processing and their interactions during monolingual reading [3]. By contrast, evidence remains limited for bilingual task contexts such as translation and post-editing (PE), in which reading is coupled with cross-language reformulation. Reading for translation appears to differ from within-language reading as it takes more working memory resources and may involve activation of target language lexical entries [4].

Translation is a form of bilingual text production in which comprehension of the source text is closely tied to production of the target text [5]. Translators do not first finish reading and then begin writing; rather, they move back and forth between comprehension, formulation, and monitoring as the target text is incrementally produced [6,7]. Eye movements in translation should therefore reflect not only text understanding, but also the demands of bilingual text production.

Post-editing differs from translation in one key respect. Although both tasks require readers to coordinate source and target text, post-editing additionally involves evaluating, verifying, and selectively revising the draft [8,9]. Therefore, translation and PE should be treated neither as monolingual reading nor as the same form of bilingual reading [10].

Multilingual pre-trained models offer a novel way to quantify reading activity during both tasks. Prior work has modeled human translation (HT) difficulty using surprisal, entropy, and neural predictors derived from corpus or deep neural models [11,12,13]. In this study, we focus on two model families that differ in architecture, decoder-only language models (LMs) and encoder–decoder neural machine translation (NMT) models. LM predictors capture predictability within a language through monolingual surprisal [14,15], whereas NMT predictors capture uncertainty with source context and target predictions [13].

It remains unclear, however, whether these predictors relate to translators’ eye movements across source and target reading and whether such relations vary by task type or translation direction. The present study is guided by three research questions.

- RQ1: Do translators’ reading measures align more strongly with LM surprisal or NMT predictors?
- RQ2: Does the relationship between predictors and reading behavior vary across translation directions?
- RQ3: Do human translation (HT) and post-editing (PE) differ in the relation between predictors and reading behavior?

## 2. Related Work

### 2.1. Eye Movements Across Reading Tasks

Eye movements provide evidence of ongoing processing during reading. In monolingual reading, measures such as first-fixation duration, gaze duration, and regressions have been used to characterize lexical access, sentence integration, and oculomotor control [16]. Early measures are often interpreted as more sensitive to relatively early stages of word processing, whereas later measures such as total reading time and regressions are more informative about later integration difficulty, monitoring, and rereading [17].

Eye movement patterns, however, are not always consistent across reading situations. Task demands can alter both temporal and spatial aspects of reading behavior. For example, proofreading and reading for comprehension lead to different fixation and saccade patterns [18]. At the page level, thorough reading, skimming, and spell-checking also produce different patterns [19].

In bilingual reading, L2 differs from L1 reading, with L2 involving longer fixation durations and more frequent regressions [20]. Lexical processing in bilingual reading can be influenced by cross-language activation even in strongly unilingual sentence contexts [21]. Moreover, evidence on parafoveal processing in bilingual readers suggests that cross-language semantic information is not accessible during reading in a monolingual language mode, consistent with the partial selectivity hypothesis of bilingual language control [22]. These findings highlight that bilingual reading behavior depends not only on general L1 or L2 proficiency differences but also on task-level language mode.

### 2.2. Translation and Post-Editing as Bilingual Reading

Bilingual comprehension and production are closely coordinated during translation [6,7]. Rather than executing sequential steps, translators simultaneously consult the source text and plan target output [5]. Recent work suggests that non-literality, lexical or morphological restructuring, and compound handling can redistribute effort across source reading, target reading, and production duration [10,23]. Eye-tracking and key-logging studies further show that L1→L2 translation places greater demands on target text production while L2→L1 translation draws more heavily on source text comprehension [24], and that preparatory reading patterns vary by both translation mode and direction [25].

### 2.3. Surprisal, Reading Time, and Model Fit

Rooted in information theory, surprisal quantifies how unexpected a token is given its preceding context. It has been proposed as a measure of processing difficulty [14,15]. Higher surprisal is often associated with slower reading [26,27,28,29]. In practice, it has been estimated using n-gram models [30], probabilistic grammars [14], and more recently large language models (LLMs) [28,31]. The mapping from model quality to predictive power is, however, non-trivial, since perplexity does not always produce good fits to human processing [31,32]. Training experiments show that Transformer-based LM surprisal aligns best with human reading times after about two billion training tokens; past a certain threshold, however, additional training data cause surprisal estimates to diverge from expectations, inverting the scaling relationship between model size and its fit to human reading [33]. Recent evidence further indicates that model fit improves when models better match readers’ experience and task ecology [34], and that text difficulty can modulate predictability effects beyond what average surprisal accounts for [35].

Recent work has also explored whether LLMs can serve as cognitively plausible models of human language processing. A central finding is that larger models do not necessarily produce better predictions of human reading behavior; rather, internal representations from intermediate layers often align better with human measures than final-layer predictions do [36]. This layer-wise correspondence suggests that different processing stages in neural LMs may map onto different aspects of human cognition, with early layers approximating incremental parsing and later layers capturing more integrated comprehension [37].

Beyond reading times, LLMs have been shown to predict neural correlates of language processing. Studies using EEG have demonstrated that LLM internal representations, particularly from middle layers, correlate with N400 potentials [36]. Similarly, research on spontaneous speech production suggests that fine-tuned LLMs can capture production variables such as speech reduction and prosodic prominence that reflect cognitive processing load [38].

The cognitive plausibility of LLMs also depends on the training mechanism and dataset. Models trained on spoken language corpora show stronger alignment with human production measures than those trained exclusively on written text [38], underscoring the role of ecological validity; recent work demonstrates that LLMs fine-tuned on sentence-level psycholinguistic norms can predict human reading times better than baseline predictors, though zero-shot performance remains inconsistent [39].

### 2.4. Multilingual Pre-Trained Models in Translation Difficulty Modeling

Model architecture has been shown to affect the predictive power of surprisal for human reading behavior, independent of perplexity [31,32]. Decoder-only and left-to-right LMs provide monolingual next-token predictability. By contrast, multilingual encoder–decoder NMT models estimate target probabilities conditioned on source context and therefore offer translation surprisal [13,40]. Prior studies show that NMT surprisal and derived features can explain variation in human translation difficulty [12,13]. Attention and entropy descriptors may add complementary information beyond surprisal [41,42], but attention weights should be interpreted as internal variables of the model rather than direct measurements of human attention [41,43].

### 2.5. Research Gap and Positioning of the Present Study

Several questions remain unresolved. First, while surprisal theory has been extensively tested in monolingual reading, it remains unclear whether monolingual LM surprisal or bilingual NMT translation surprisal better captures processing difficulty during translation, where comprehension and production are interleaved across languages. Second, the ongoing debate about whether LM surprisal effects reflect genuine contextual prediction or frequency confounds [44] has not been extended to bilingual task settings, where NMT predictions offer a qualitatively different predictability measure. Third, prior translation difficulty modeling has focused on human translation alone [13], leaving open whether post-editing, which adds evaluation and revision to the production process, changes the mapping between predictability and behavioral measures or merely reduces mean effort. The present study attempts to address these gaps by jointly modeling source reading, target reading, and target production duration in a within-subject design.

## 3. Materials and Methods

### 3.1. Participants, Materials, and Apparatus

We designed a within-subject experiment comparing task type (translation vs. post-editing) and direction (en→zh vs. zh→en). Drafts for post-editing were generated with gpt-4o-2024-05-13 [45], and 26 Chinese L1 student translators (*M*_age_ = 22.96, *SD* = 1.40) were recruited from a university in central China. Eye movements were recorded with an EyeLink 1000 Plus in remote mode at 1000 Hz, with 13-point calibration performed before each task. The display was configured at 1280 × 1024 pixels and 75 Hz refresh rate. User activity data were synchronized via Translog-II [46].

**Figure 1:**
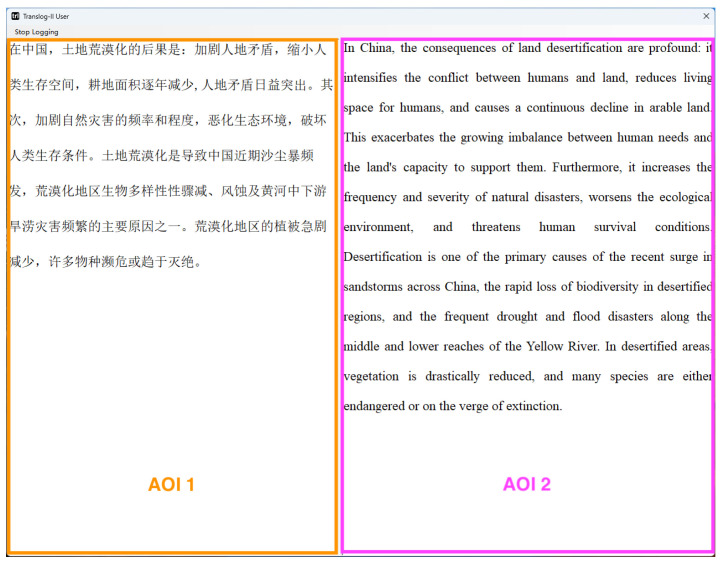
Screen layout in Translog-II showing the two AOIs. AOI 1 covers the source text region; AOI 2 covers the target text region. Eye-tracking measures were extracted from two AOIs.

We used a horizontal split-screen layout with source text positioned left and target text right. Chinese text used SimSun font and English text used Times New Roman, both at 18-point size with double line spacing. Figure 1 illustrates the screen layout and the two defined Areas of Interest (AOIs).

Task materials comprised four non-literary texts drawn from English–Chinese translation examinations [47]. They are suitable for practical translation testing [48]. Each task was kept to approximately 85 words, with Chinese word counts computed after tokenization. The order of passages was randomly assigned to participants.

To match the difficulty of source materials, the readability of each task material was assessed by mdeberta-v3-base-readability, a fine-tuned model capable of estimating readability scores across languages [49]. Scores estimated by the model were closely clustered across texts, ranging from 19.06 to 19.86, with condition means of 19.38 for translation and 19.54 for post-editing materials. As an additional check, three professional translators (mean experience: 5.3 years) independently rated each text on a 10-point difficulty scale (1 = very easy, 10 = extremely difficult). As shown in Table 1, human ratings broadly aligned with the automated scores, suggesting that the difficulty of the materials was comparable.

### 3.2. Data Filtering and Alignment

Gaze data were aligned so that fixations could be mapped onto source and target units. At the token level, each unit corresponds to one aligned source or target token; at the segment level, aligned tokens are merged into one unit. In the aligned tables, TrtS and TrtT capture reading behavior, while Dur was treated as a production measure.

We applied separate filtering criteria to each measure. For TrtS and TrtT, values below 20 ms were excluded, following previous work [13,50]. Because post-editing sessions may contain zero values in target duration for unedited units, Dur was modeled only when it was positive and at least 20 ms. All retained continuous values were log transformed before modeling.

Under these filters, source TrtS covered 89.8% of token-level observations and 90.9% of segment-level observations; target TrtT covered 90.4% of token-level observations and 92.2% of segment-level observations; positive target Dur covered 52.6% of target tokens and 53.9% of target segments. Two post-editing sessions (zh→en) were excluded from token- and segment-level analyses because heavy structural editing during post-editing broke the source–target token alignment; these sessions contributed only sentence-level observations. The full experimental dataset comprises 104 task sessions.

### 3.3. Model Selection and Feature Extraction

We selected two pre-trained models to compute the predictors of processing difficulty. mGPT [51], a multilingual causal language model, generates left-to-right surprisal estimates for each language independently, computed as token-level negative log-probabilities over sentence sequences. NLLB-200 [40], a multilingual NMT model, provides probabilities with source context alongside encoder, decoder, and cross-attention tensors. Masked language models such as BERT [52] were not considered, as they rely on bidirectional context and do not natively produce left-to-right sequence probabilities. The resulting estimates are pseudo-likelihoods [53,54], and therefore do not yield surprisal values equivalent to those of causal language models.

**Figure 2:**
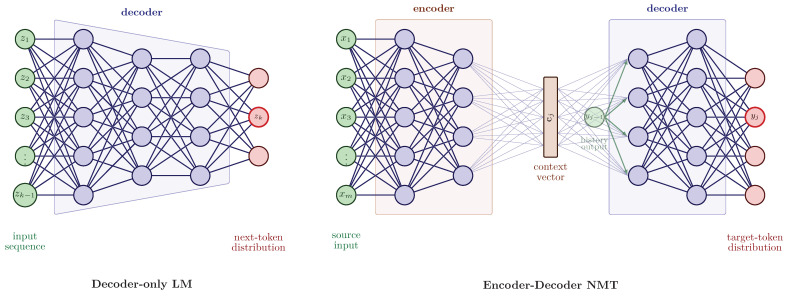
Architecture comparison of the two model types used in this study. (**Left**): A decoder-only LM maps input sequence to a next-token distribution. (**Right**): An encoder–decoder NMT model encodes $x_{1:m}$ and combines that source representation with the previous target tokens $y_{<j}$ to predict $y_{j}$.

As illustrated in Figure 2, two processing difficulty predictors can be derived from these two architectures. The first is monolingual surprisal, the negative log probability of a token given its preceding context, provided by decoder-only LMs. The second is translation surprisal, which quantifies the uncertainty in target generation conditioned on both the source sequence and preceding target context, derived from the encoder–decoder NMT model [13].

Formally, let $x_{1:m}$ be a source sequence and $y_{1:n}$ a target sequence. Monolingual surprisal on the source side is

(1) $$S_{\mathrm{lm}}\left(u\right)=\sum_{i\in u}-\log p_{\mathrm{lm}}\left(x_{i}|x_{<i}\right),$$

and for monolingual surprisal in the target text

(2) $$S_{\mathrm{lm}}\left(v\right)=\sum_{j\in v}-\log p_{\mathrm{lm}}\left(y_{j}|y_{<j}\right).$$

**Figure 3:**
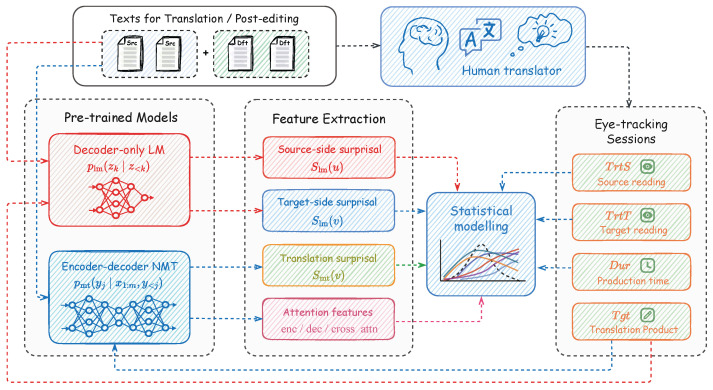
Overview of the modeling framework. Model-derived predictors and attention features are compared with measures from eye-tracking sessions.

For the encoder–decoder NMT model, translation surprisal is

(3) $$S_{\mathrm{mt}}\left(v\right)=\sum_{j\in v}-\log p_{\mathrm{mt}}\left(y_{j}|x_{1:m},y_{<j}\right).$$

where *u* and *v* are source and target token sets, respectively. At the token level, *u* and *v* are singletons. At the segment level, *u* and *v* denote merged aligned units, and segment-level surprisal is the sum of the token-wise surprisals inside that aligned unit. Attention features, originally computed per token, are averaged across tokens within the same aligned unit before segment-level modeling. Models of source reading use $S_{\mathrm{lm}}\left(u\right)$, while models of target reading include both $S_{\mathrm{lm}}\left(v\right)$ and $S_{\mathrm{mt}}\left(v\right)$. The modeling framework is summarized in Figure 3.

For other attention feature extraction, we followed prior work [13] and stacked attention tensors across all layers and heads from the encoder self-attention, cross-attention, and decoder self-attention, and computed two types of scalar features per aligned unit. Coverage features (*f*) measure the summed attention mass from one set of positions to another, for example, encoder self-attention from a source token to its surrounding context ($f_{u,\bar{u}}^{e}$) or cross-attention to the sequence boundary ($f_{v,\mathrm{eos}}^{c}$). Entropy features (*H*) capture the diffuseness of the attention distribution, with higher values indicating more evenly spread attention. Coverage and entropy features were normalized by the corresponding uniform-attention baseline before being averaged across layers and heads. A complete feature glossary is provided in Appendix A, Table A1, Table A2, Table A3 and Table A4.

### 3.4. Model Estimation and Statistical Inference

For each combination of side, aggregation level, and outcome, we fitted regression models that account for the structure of the data. The continuous reading outcomes were modeled with linear mixed-effects models (LMMs). The fixed-effect structure compared a control model with an augmented model containing one computational predictor:

(4) $$\begin{array}{cc} y∼ & \mathrm{length}+\mathrm{frequency}+\mathrm{position}\\ \; & +\mathrm{predictor}+C(\mathrm{lang}\_\mathrm{pair}). \end{array}$$

Here, length, frequency, and position are segment length, mean unigram frequency, and within-sentence position quantile of the modeled unit. Models of source reading use source-side controls, whereas target reading and duration models use target-side controls. Intercept-only models can yield anticonservative inferences in repeated-measures designs, so we used the maximal random-effects structure [55]. For each comparison, we first fitted the correlated maximal structure justified by the design, with crossed by-participant and by-item random intercepts and correlated by-participant and by-item random slopes for the focal predictor, (1+predictor∣participant)+(1+predictor∣sentenceitem). When this model failed to converge or returned a singular fit, we applied a stepwise recovery strategy: we first removed the random-effect correlations, then dropped the by-item slope, and finally fell back to random intercepts only, retaining the most complex structure that fit cleanly. Random slopes were specified for the focal predictor; by-item slopes for task type or translation direction were not included because sentence items were nested within task and direction and such slopes are therefore not estimable. When even the random-intercept model produced a boundary fit, it was retained only where the boundary reflected a near-zero random-effect variance rather than a failure of fixed-effect estimation; these cases are flagged in the convergence diagnostics (Appendix B) and interpreted cautiously. For every comparison, the augmented model was matched to a control model with the same random-effects structure, so the likelihood-ratio test evaluated only the added fixed-effect of the focal predictor (df=1). Across the 666 main-effect and residual attention comparisons, 256 used the correlated maximal structure, 85 the uncorrelated maximal structure, 78 the by-participant-slope structure, and 247 the random-intercept structure; no fit was discarded.

Continuous values were log transformed and standardized prior to fitting. Model improvement was quantified by comparing maximum-likelihood fits for the augmented and control models:

(5) ΔLL=ℓ(control+pred)−ℓ(control).

A positive ΔLL indicates that the predictor improves model fit beyond the matched control baseline. We report ΔLL normalized by the number of observations for comparability across outcome conditions. Because ΔLL is not comparable across predictors fitted under different random-effects structures, we rank predictors within each outcome by their standardized fixed-effect coefficient $\hat{\beta }$, each estimated under the maximal structure where it converged and under the reduced structure otherwise, and use ΔLL only to test whether a given predictor improves fit over its matched baseline. For inference, raw *p*-values were obtained from likelihood-ratio tests ($\chi^{2}$, df=1) comparing the augmented and control models. These *p*-values were then adjusted with the Benjamini–Hochberg false discovery rate (FDR) procedure [56] and reported as *q*. Results with q<0.05 are treated as significant. The Benjamini–Hochberg procedure was applied within three families: the 666 main-effect and residual attention comparisons, the 84 interaction comparisons, and the 252 two-part duration comparisons (edit occurrence and conditional duration). All models were fitted in R [57] with the lme4 package [58].

Post-editing duration required an additional model because zeros in Dur are meaningful as they indicate that an aligned unit did not trigger editing or typing. We therefore treated Dur as a two-part process, following the two-part model framework for semicontinuous outcomes [59]. In the first part, a logistic generalized linear mixed model (GLMM) was fitted for edit occurrence, edited=1 if Dur>0 and 0 otherwise:

(6) $$\begin{array}{cc} \mathrm{edited}∼ & \mathrm{length}+\mathrm{frequency}+\mathrm{position}+\mathrm{predictor}\\ \; & +C(\mathrm{lang}\_\mathrm{pair})+(1+\mathrm{predictor}|\mathrm{participant})+(1+\mathrm{predictor}|\mathrm{sentence}\;\mathrm{item}). \end{array}$$

Second, for units that were actually edited, we fitted the positive duration LMM described above. Both parts of the two-part model used the same maximal-then-reduced random-effects specification as the reading models, with by-participant and by-item random slopes for the focal predictor reduced on nonconvergence or singularity.

To test whether predictor–behavior relations varied across task types or translation directions, we fitted pooled additive and interaction mixed models. The additive model included the control terms, the predictor, task type, and language direction; the interaction model additionally included either predictor×C(task_type) or predictor×C(lang_pair). The additive and interaction models shared the same random-effects structure—a by-participant random slope for the focal predictor with a by-item intercept, initially fitted as correlated, then reduced to uncorrelated, and finally to random intercepts only on nonconvergence or singularity. By-item slopes for the focal predictor were not added here because the interaction models with that structure rarely converged, and by-item slopes for task type or direction are not estimable given the nesting of items within task and direction. The interaction contrast was defined as:

(7) $$\Delta LL_{\mathrm{int}}=\ell \left(\mathrm{interaction}\;\mathrm{model}\right)-\ell \left(\mathrm{additive}\;\mathrm{model}\right).$$

## 4. Results

### 4.1. Predictors of Reading Time and Production Duration

**Figure 4:**
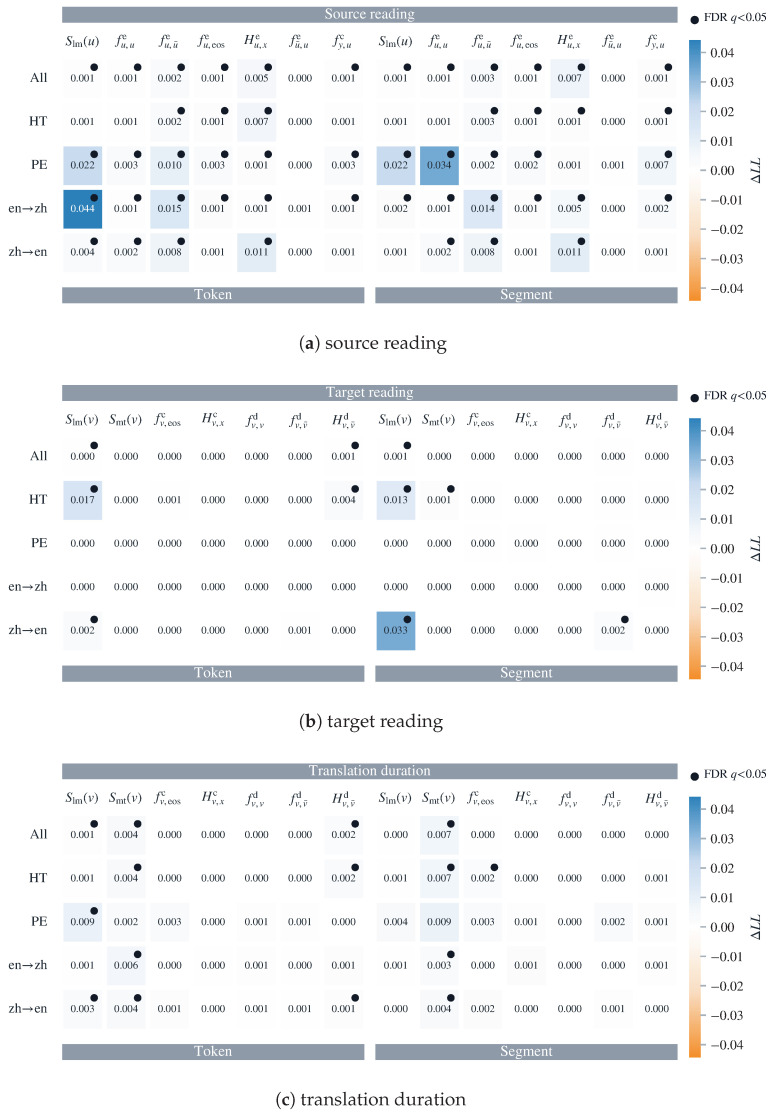
ΔLL relative to matched control baselines for source reading, target reading, and target duration. Rows summarize the full dataset, task subsets, and direction subsets.

Source reading is most strongly associated with encoder-side attention, together with source text predictability. At both token and segment levels, the encoder self-attention feature $f_{u,u}^{e}$ carries the largest standardized coefficient, while source LM surprisal $S_{\mathrm{lm}}\left(u\right)$ remains a robust positive predictor that significantly improves fit. For target reading, target LM surprisal $S_{\mathrm{lm}}\left(v\right)$ is the strongest predictor at both levels; NMT translation surprisal does not reach significance. For positive Dur, $S_{\mathrm{lm}}\left(v\right)$ is strongest at the token level, whereas at the segment level, NMT translation surprisal $S_{\mathrm{mt}}\left(v\right)$ is the strongest significant predictor and $S_{\mathrm{lm}}\left(v\right)$ is comparable in magnitude ($\hat{\beta }=0.084$) but does not survive FDR correction (Table 2). Predictors are ranked by their standardized coefficient $\hat{\beta }$, each estimated under the maximal structure where it converged and under the reduced structure otherwise; ΔLL/n and *q* index indicate whether that predictor improves fit over its matched baseline. Figure 4 extends this comparison with a breakdown by task type and translation direction.

**Figure 5:**
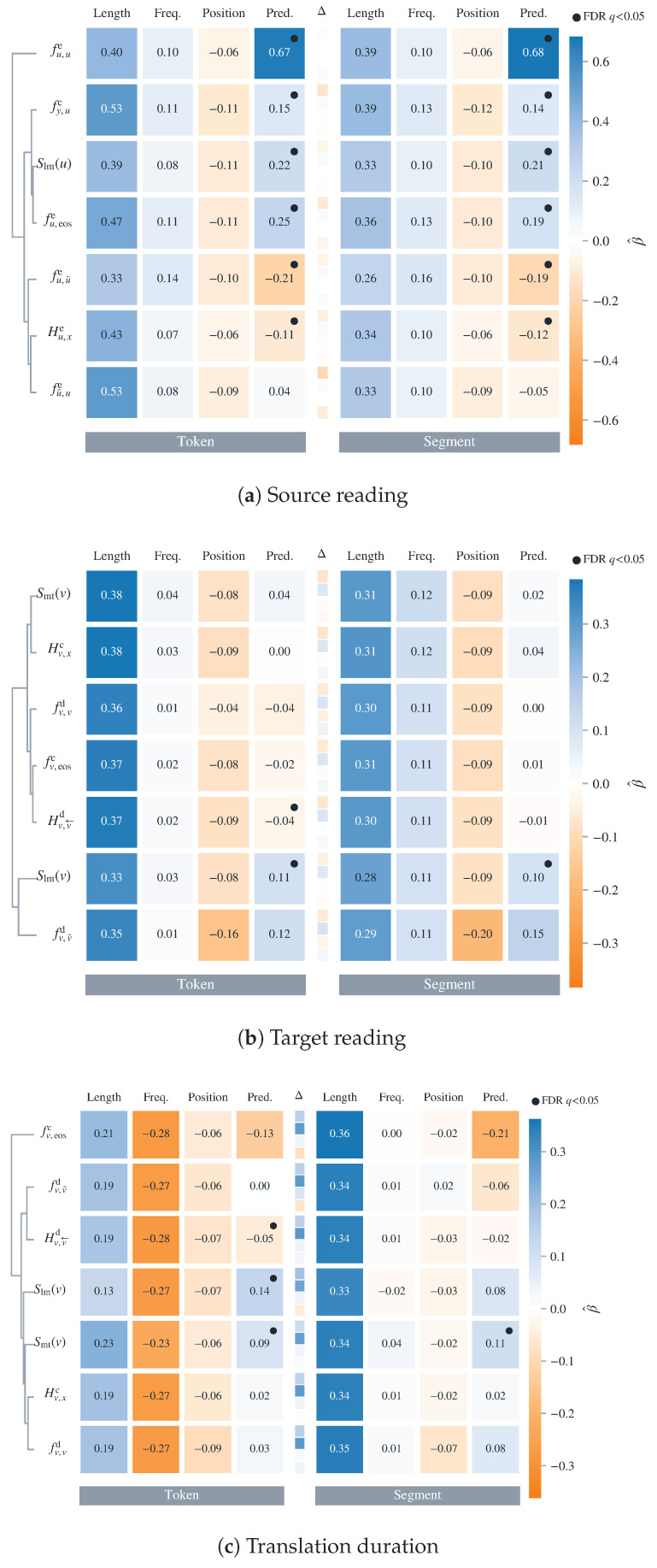
Coefficient estimates for TrtS, TrtT, and Dur. Each heatmap shows the control terms and the predictor for token- and segment-level models.

**Figure 6:**
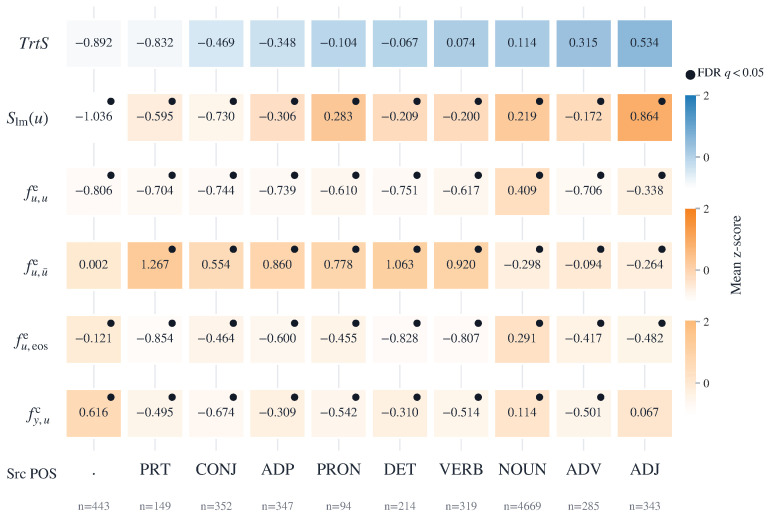
POS summaries for source reading. Larger values indicate POS categories for which model-derived predictors explain more variance in source reading time.

**Figure 7:**
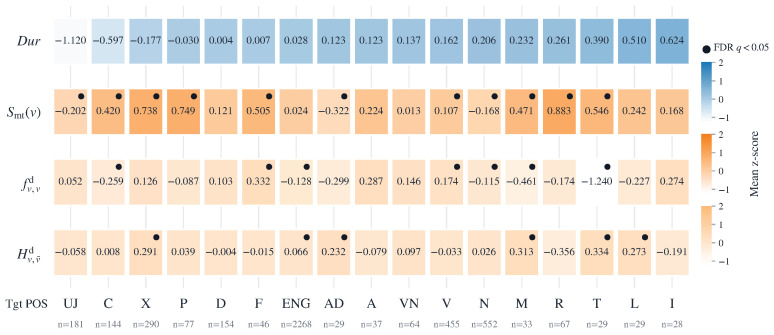
POS summaries for target reading and duration. The target-side pattern is more dispersed than the source-side pattern, consistent with the mixed reading and production character of target behavior.

The coefficient estimates in Figure 5 show that these predictors retain interpretable effects after controlling for segment length, word frequency, and within-sentence position, under the maximal random-effects structure described above. A part-of-speech breakdown further reveals that source-side predictor effects were largest for content words (adjectives, nouns), while target-side effects were more evenly distributed across POS categories, reflecting the mixed reading and production processes that target duration captures (Figure 6 and Figure 7).

**Figure 8:**
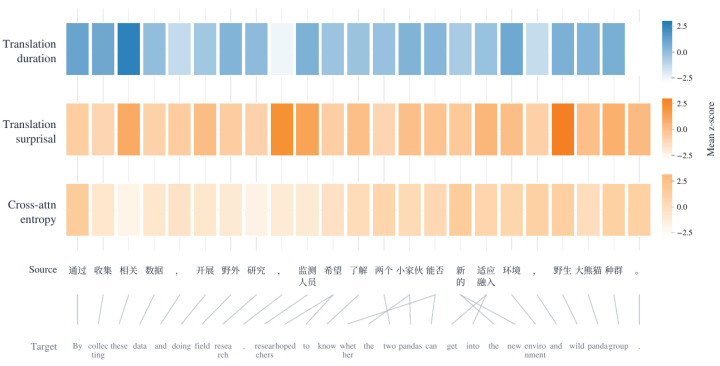
Aligned unit example comparing standardized human duration, translation surprisal, and cross-attention entropy.

Figure 8 illustrates the alignment between source and target tokens for an example sentence, showing how standardized duration, translation surprisal, and cross-attention entropy map onto a common source–target alignment.

### 4.2. Task, Direction, and Interaction Effects

**Figure 9:**
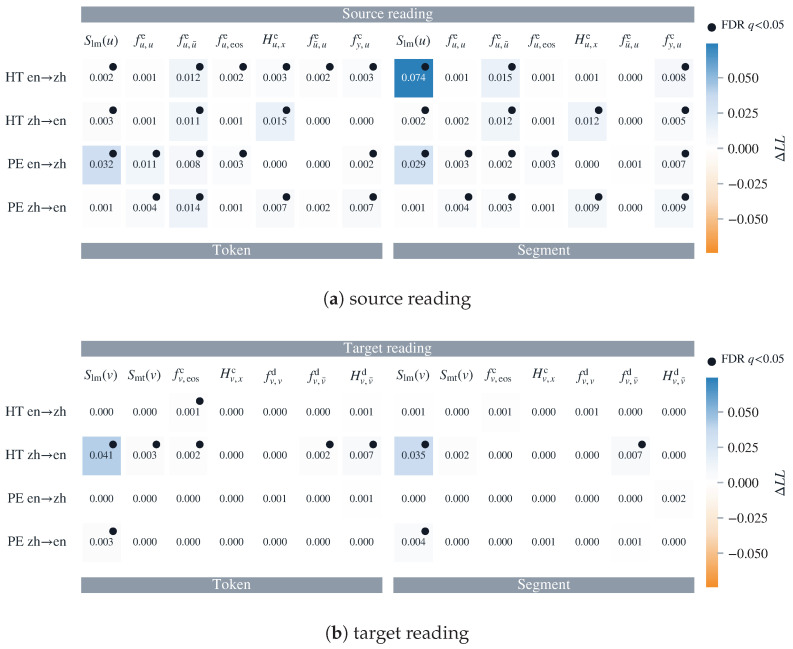

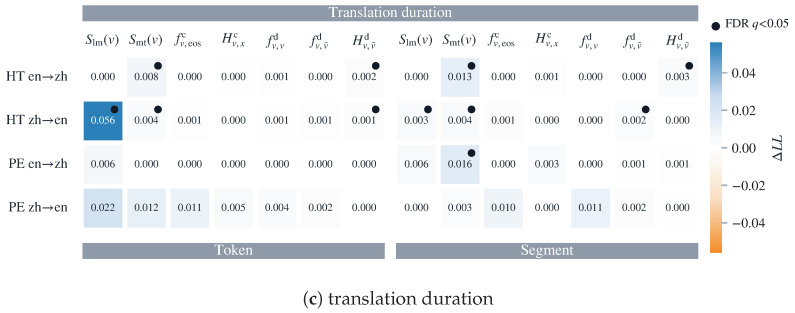
ΔLL/n in the four task-by-direction conditions: translation en→zh, translation zh→en, postediting en→zh, and post-editing zh→en.

The grouped summaries show that direction produces broader changes than task type (Table 3). en→zh has stronger source reading effects, whereas zh→en has stronger target reading effects; duration effects are comparable across directions. Because the direction split is so pronounced, target-side effects can be larger within a single direction or task than in the pooled sample, where opposing directional patterns partly cancel. Translation and post-editing differ most clearly on target-side measures, but post-editing does not eliminate predictor effects: source reading remains highly predictable in this task, even though target reading predictors lose significance there. Figure 9 shows the full predictor-level breakdown across the four task-by-direction conditions.

**Figure 10:**
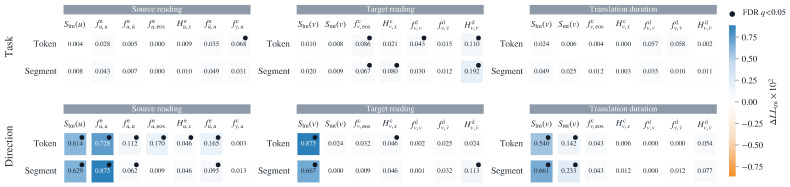
Predictor × task (**top**) and predictor × direction (**bottom**) interaction. $\Delta LL_{\mathrm{int}}$ measures improvement from adding the interaction term to the additive mixed model.

The pooled interaction models show that direction modulates predictor effects more broadly than task type: direction interactions reach FDR significance for 18 of 42 predictor comparisons, and task interactions for 7 of 42. The largest direction interactions involve $f_{u,u}^{e}$ for source reading and $S_{\mathrm{lm}}\left(v\right)$ for both target reading and duration. Task interactions, although fewer, are not uniformly absent: they reach significance chiefly on target reading, where decoder self-attention entropy $H_{v,\bar{v}}^{d}$ interacts with task type at both levels (Table 4; Figure 10).

### 4.3. Temporal Decomposition of Duration

**Figure 11:**
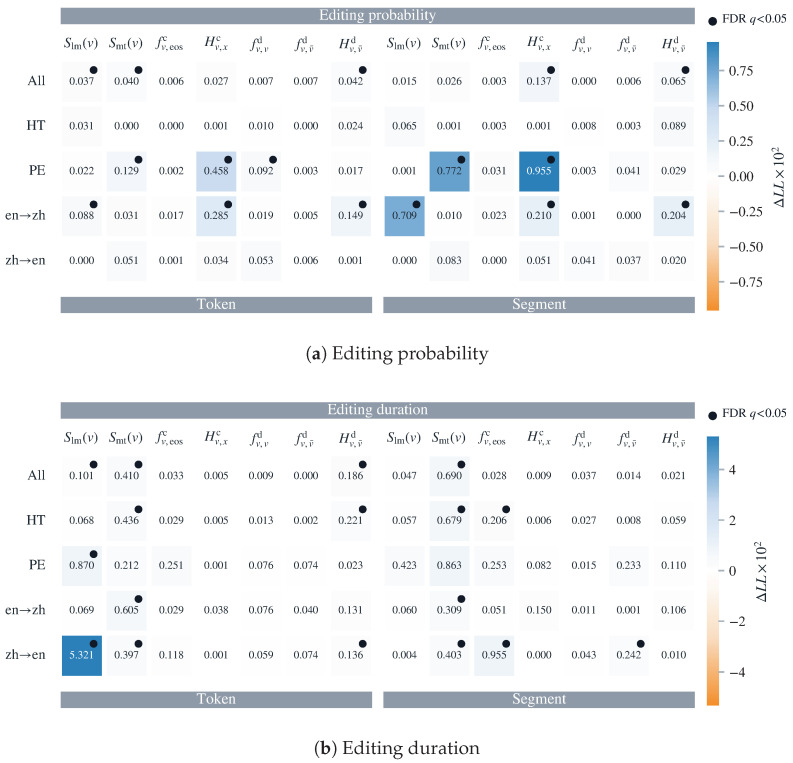
Two-part duration model. (**a**) Edited or not (logistic GLMM); (**b**) edit duration (LMM).

Duration is a production measure that complements the reading measures, but the behavioral processes are not directly observable from gaze. To extend the temporal window from gaze to behavioral response, we treated target duration as a two-stage process. Most aligned units receive zero duration because no gaze was allocated or no editing was triggered. Positive durations reflect how long production persists once initiated. The two-part model therefore decomposes the temporal process into two stages: edit occurrence and conditional editing duration. In the editing probability model, edit occurrence was predicted by a subset of target-side features. Decoder self-attention showed the clearest pattern, while cross-attention contributed mainly at the segment level; LM and NMT surprisal also reached significance in selected token-level comparisons (five of fourteen token/segment comparisons reached significance). A supplementary comparison of features computed from the original drafts used for post-editing showed a direction-dependent pattern: the features distinguished edited from unedited segments in one post-editing text but not the other. In the conditional editing duration model, NMT translation surprisal was the strongest predictor of editing time among units that were actually edited, with target LM surprisal and decoder self-attention entropy also significant (Figure 11).

**Figure 12:**
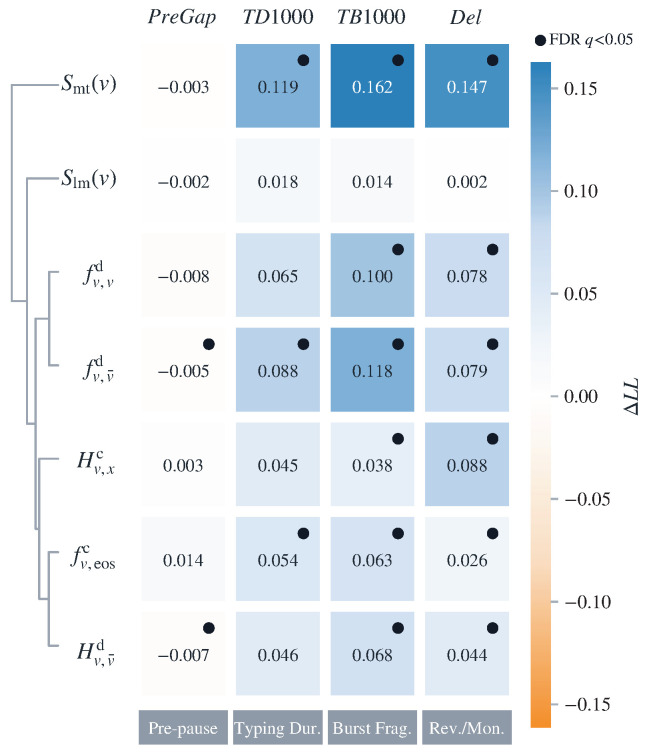
Key-logging ΔLL for target duration decomposition.

To further interpret the duration component, we decomposed positive duration into four key-logging measures from the CRITT TPR-DB segment-level variables [6]: pre-pause (*PreGap*, the pause duration preceding the first keystroke, which we label as *Pre-pause*); typing duration (*TD1000*, the production time within a segment excluding pauses longer than 1000 ms between keystrokes, which we label as *Typing Dur.*); burst fragmentation (*TB1000*, the number of continuous keystroke sequences within a segment separated by pauses longer than 1000 ms, which we label as *Burst Frag.*); and revision or monitoring (*Del*, the number of deleted characters, which we label as *Rev./Mon.*). These measures map the temporal process onto specific motor and cognitive operations. NMT translation surprisal $S_{\mathrm{mt}}\left(v\right)$ is the leading predictor of typing duration, burst fragmentation, and deletion effort, while decoder-side attention features contribute across all four measures, with planning pauses showing a distinct negative association with decoder continuation features (Figure 12). This decomposition indicates that production duration reflects multiple distinct behavioral processes.

### 4.4. Residual Contribution of Attention Features

**Figure 13:**
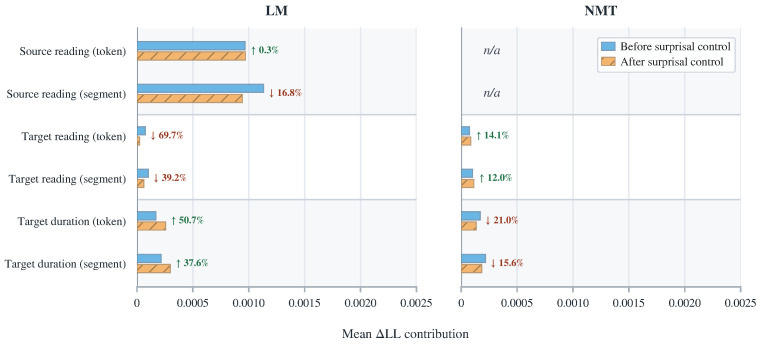
Paired comparison of mean attention feature ΔLL before and after surprisal control. Arrows indicate the relative change in ΔLL contribution.

To quantify how much attention features contribute beyond surprisal, we define *A* as the log-likelihood gain from adding attention features to a baseline model, with $A_{0}=\Delta LL\left(\mathrm{attention}|\mathrm{controls}\right)$ computed before surprisal included and $A_{1}=\Delta LL\left(\mathrm{attention}|\mathrm{controls}+\mathrm{surprisal}\right)$ computed after. To keep $A_{0}$ and $A_{1}$ on a comparable scale, both were computed under the same random-effects structure for a given feature, and Figure 13 plots $A_{0}$ against $A_{1}$ for the features that reached the maximal structure. In source reading, the attention contribution stays positive after source surprisal is included: it is essentially unchanged at the token level (from 0.00097 to 0.00097) and decreases at the segment level (from 0.00113 to 0.00094). Target-side attention effects are substantially smaller overall. For target duration, $A_{1}$ is comparable to or exceeds $A_{0}$ for some predictors, suggesting that target production and revision contain structure beyond what surprisal captures.

**Figure 14:**
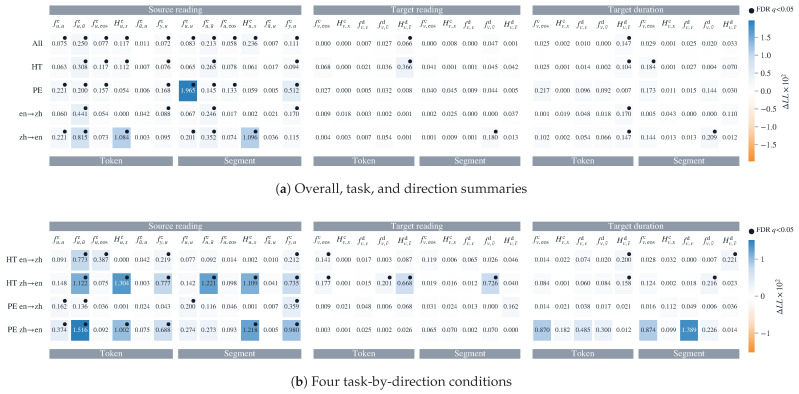
Residual attention ΔLL after controlling for surprisal. (**a**) Overall, task, and direction summaries, with values scaled by $10^{2}$; (**b**) four task-by-direction conditions.

The residual attention patterns further differ by task and direction (Figure 14). Source-side attention effects remained positive after surprisal control, whereas target-side attention contributions were smaller and more condition-dependent. For target duration, the largest residual attention values appeared descriptively in zh→en post-editing, but these effects should be interpreted as exploratory because they did not reach FDR significance.

## 5. Discussion

### 5.1. Monolingual Predictability in Bilingual Reading

The pattern across outcomes suggests a division of labor between monolingual expectancy and bilingual generation uncertainty. Cumulative reading measures were mainly aligned with LM-based predictability, especially for target reading, whereas target production duration showed stronger sensitivity to NMT translation surprisal when target units were aggregated at the segment level. Source reading occupied an intermediate position: encoder self-attention focus $f_{u,u}^{e}$ was the strongest predictor, while source LM surprisal remained a robust secondary predictor. This dissociation indicates that translators’ eye movements are not simply tracking source-conditioned translation uncertainty. Instead, cumulative reading appears to reflect incremental expectancy within the currently processed text stream, whereas source-conditioned uncertainty becomes more visible when reading is coupled with target production and revision. This interpretation is consistent with the incremental nature of eye movement processing, in which fixation durations reflect difficulty during reading [1,17], and with surprisal accounts in which processing cost scales with predictability [14,15,27].

These findings do not imply that translation reading is functionally monolingual. Cross-language co-activation during bilingual word recognition can be modulated by task context [60]. Evidence from bilingual sentence reading likewise suggests that lexical access is not fully language-selective, as shown by cognate facilitation even in a strongly unilingual sentence context [21]. Reading for translation requires more working memory resources than reading for repetition [4], and target language lexical and syntactic properties can be activated during source language processing [61]. These results refine earlier work: translation reading remains bilingual in task structure and available lexical activation, but cumulative reading times are more strongly indexed by monolingual predictability than by translation uncertainty.

### 5.2. Direction, Task, and Post-Editing Duration

Direction effects are broader than task effects in both the grouped summaries and the pooled interaction models. This suggests that relations between predictor and reading behavior change more between L1→L2 and L2→L1 than between translation and post-editing alone, consistent with bilingual eye movement research showing that L2 reading typically involves longer reading times and more effortful eye movement patterns than L1 reading [20]. This also fits evidence that lexical frequency effects are stronger in bilingual L2 reading than in L1 reading [62]. Translation direction therefore appears to be more than a difference in overall task difficulty. It appears to shape which predictors are reflected in behavior and how processing demands are distributed across source processing, target formulation, and monitoring.

Task effects were narrower than direction effects but not entirely absent: task interactions reached significance for fewer predictor comparisons than direction interactions, and were concentrated on target reading, where decoder self-attention entropy interacted with task type. This indicates that HT and PE differ only in selected respects in how predictors map onto reading and production measures. Studies of reading have long shown that eye movement behavior varies with task demands, including contrasts among careful reading, comprehension-oriented reading, proofreading, and skimming [18,19]. The comparatively limited task interactions here suggest that translation and post-editing may draw on a largely shared bilingual reading architecture, which would explain why they pattern similarly across most measures.

Post-editing, however, changes the role of the target text: it is not only produced, but also inspected, diagnosed, and selectively edited. The two-part Dur analysis makes a related but distinct point. A target unit must first trigger editing, and only then can positive editing duration be observed. This distinction, between whether and how long editing occurs, operates within the duration measure itself, rather than as a task-level interaction with predictor effects. Under the maximal random-effects specification, edit triggering was predicted by a subset of target-side features, mainly decoder self-attention entropy and surprisal, so both stages are sensitive to model-derived predictability rather than only the conditional duration stage, although edit occurrence is captured more selectively than editing duration. This yields a methodological implication: Dur should not be interpreted as a single continuous proxy for production effort. In post-editing, it combines at least three separable processes: whether editing is triggered, how long production continues once triggered, and how revision unfolds through typing bursts, deletions, and pauses. Key-logging results support this interpretation by showing that target duration draws on typing duration, burst fragmentation, deletions, and pre-pause time rather than a single effort dimension. This fits evidence that post-editing effort varies across indicators [63], depends on MT error type [64], and may diverge across product and process measures [65]. Weaker or more selective predictor links in post-editing should not be taken as evidence of reduced cognitive involvement; a more plausible interpretation is that revision-dominated behavior is less well captured by predictors derived from continuous generative sequences.

### 5.3. Attention Features and Theoretical Implications

The attention results are clearer under this interpretation. Attention-derived features did not replace surprisal as the primary predictor, but they helped identify where model-internal structure remains behaviorally informative after expectancy is controlled. Their contribution was clearest for source reading and for post-editing target duration, suggesting attention-derived features are best interpreted here not as direct analogs of human attention, but as diagnostics of model-internal organization that may become relevant when translation involves alignment, monitoring, or revision. This interpretation is consistent with work showing that transformer attention patterns can contribute incremental predictive value for reading times [41,42], while also respecting cautions that model attention weights are internal allocation variables rather than transparent explanations of model behavior [66,67]. NMT attention may help explain model predictions while still failing as a straightforward alignment between source and target tokens [43]. Surprisal, by contrast, provides a more reliable link between probabilistic language modeling and cumulative eye movement behavior.

Taken together, these results suggest a graded, reweighted processing system for translation reading. Translators do not switch between a purely monolingual mode and a purely bilingual mode. Rather, bilingual task demands remain available throughout processing, while direction and task type continuously reweight the balance between source comprehension, target formulation, and monitoring. Within that shifting system, cumulative eye movement measures are primarily indexed by incremental monolingual expectancy, whereas bilingual translation uncertainty becomes more visible when reading is coupled with target production and revision. This pattern is also consistent with the residual attention analysis, where target-duration attention effects were numerically largest in zh→en post-editing, although they did not reach significance. This pattern suggests that revision and monitoring in L2 output editing may contain structure not fully captured by surprisal, but it should be treated as exploratory.

### 5.4. Limitations

The sample of 26 participants and 104 sessions is typical for eye-tracking translation research but still limits detailed subgroup inference; post-editing direction splits in particular should be treated as estimates with wider uncertainty. Attention features are offline correlates of model behavior rather than online causes of reading behavior, and all effects reflect predictive contrasts rather than causal estimates. The between-text design remains a limitation for interpreting direction and task effects; future studies would benefit from designs that pair larger participant samples with a shared, controlled set of materials.

## 6. Conclusions

This study examined how predictors derived from multilingual pre-trained models account for translators’ eye movements during translation and post-editing across two translation directions. The findings reveal a functional division between monolingual predictability and bilingual translation uncertainty. Cumulative reading measures were primarily linked to monolingual LM surprisal, whereas target production duration showed additional sensitivity to NMT translation surprisal, especially at the segment level. Source reading was best captured by source-side encoder focus, with LM surprisal remaining a robust secondary predictor. Translation direction further reshaped predictor–behavior relations more broadly than task type, especially on target measures, suggesting that L1→L2 and L2→L1 translation may reweight the balance between source comprehension, target formulation, and monitoring.

These findings refine how translation reading should be characterized. They do not support a simple opposition between monolingual and bilingual reading. Instead, translation appears to involve a bilingual processing ecology in which observable eye movement behavior is continuously reweighted by direction and task demands. Within this ecology, cumulative reading difficulty is more strongly indexed by incremental monolingual expectancy, while bilingual translation uncertainty becomes more visible in production and revision. The two-part duration model and key-logging decomposition further show that post-editing duration combines edit triggering, typing, burst fragmentation, deletions, and pausing rather than representing a single continuous effort measure. Attention-derived features contribute additional but smaller and more condition-dependent predictive value, best interpreted as diagnostics of model-internal organization rather than direct measures of human attention.

More broadly, the findings point toward a temporal view of translation process modeling: rather than treating source reading, target generation, and revision as separate behavioral outcomes, future models should capture these as interrelated processes unfolding over time. Future work could pursue this by deploying pre-trained models of varying scales and architectures to examine when monolingual predictability and bilingual translation uncertainty each become behaviorally relevant across this temporal sequence. Designs incorporating larger and more diverse participant samples, crossed materials, and continuous physiological measures would further allow the cognitive mechanisms of bilingual text production—and what distinguishes reading to translate from translating to read—to be traced with greater resolution.

## Figures and Tables

**Table 1:** Expert difficulty ratings (1–10 scale) for the four source passages.

Passage	Evaluator A	Evaluator B	Evaluator C	*M*
T1 (en→zh, Translation)	7.4	8.3	7.5	7.73
T2 (zh→en, Translation)	8.4	8.8	9.1	8.77
P1 (en→zh, Post-editing)	8.1	7.7	8.1	7.97
P2 (zh→en, Post-editing)	7.8	7.4	8.9	8.03

**Table 2:** Leading predictor for each outcome and aggregation level, ranked by the standardized fixed-effect coefficient $\hat{\beta }$ (95% CI) among predictors that significantly improve fit. ΔLL/n is the per-observation log-likelihood gain over the matched baseline; *p* is the likelihood-ratio *p*-value, and *q* is the FDR-adjusted value.

Outcome	Level	Leading Predictor	$\hat{\beta }$ [95% CI]	ΔLL/n	*p*	*q*
TrtS	Token	$f_{u,u}^{e}$	0.667 [0.352, 0.981]	0.00076	0.0010	0.0053
	Segment	$f_{u,u}^{e}$	0.683 [0.349, 1.017]	0.00089	0.0013	0.0067
TrtT	Token	$S_{\mathrm{lm}}\left(v\right)$	0.110 [0.035, 0.185]	0.00041	0.0109	0.0376
	Segment	$S_{\mathrm{lm}}\left(v\right)$	0.102 [0.035, 0.170]	0.00059	0.0081	0.0297
Dur	Token	$S_{\mathrm{lm}}\left(v\right)$	0.135 [0.066, 0.205]	0.00099	0.0026	0.0120
	Segment	$S_{\mathrm{mt}}\left(v\right)$	0.108 [0.078, 0.139]	0.00687	<0.0001	<0.0001

**Table 3:** Group mean ΔLL/n and number of predictors reaching FDR significance out of 14 tested token/segment combinations.

Group	TrtS	TrtT	Dur
All	0.00175 (12/14)	0.00020 (3/14)	0.00114 (4/14)
By task type			
Translation	0.00142 (7/14)	0.00264 (4/14)	0.00130 (4/14)
Post-editing	0.00788 (11/14)	0.00022 (0/14)	0.00249 (1/14)
By direction			
en→zh	0.00637 (12/14)	0.00012 (0/14)	0.00113 (2/14)
zh→en	0.00365 (7/14)	0.00275 (3/14)	0.00121 (4/14)

**Table 4:** Largest interaction effect for each outcome. Positive $\Delta LL_{\mathrm{int}}/n$ indicates improved fit relative to the corresponding additive mixed model; *p* is the likelihood-ratio *p*-value, and *q* is the FDR *p*-value.

Outcome	Level	Largest Interaction	$\Delta {\mathrm{LL}}_{\mathrm{int}}/n$	*p*	*q*
Task type interaction					
TrtS	Token	$f_{y,u}^{c}$	0.00068	0.0017	0.0076
TrtS	Segment	$f_{\bar{u},u}^{e}$	0.00049	0.0168	0.0542
TrtT	Token	$H_{v,\bar{v}}^{d}$	0.00110	<0.0001	0.0002
TrtT	Segment	$H_{v,\bar{v}}^{d}$	0.00192	<0.0001	<0.0001
Dur	Token	$f_{v,\bar{v}}^{d}$	0.00058	0.0219	0.0609
Dur	Segment	$S_{\mathrm{lm}}\left(v\right)$	0.00049	0.0661	0.1262
Direction interaction					
TrtS	Token	$f_{u,u}^{e}$	0.00728	<0.0001	<0.0001
TrtS	Segment	$f_{u,u}^{e}$	0.00875	<0.0001	<0.0001
TrtT	Token	$S_{\mathrm{lm}}\left(v\right)$	0.00874	<0.0001	<0.0001
TrtT	Segment	$S_{\mathrm{lm}}\left(v\right)$	0.00667	<0.0001	<0.0001
Dur	Token	$S_{\mathrm{lm}}\left(v\right)$	0.00542	<0.0001	<0.0001
Dur	Segment	$S_{\mathrm{lm}}\left(v\right)$	0.00661	<0.0001	<0.0001

## Data Availability

The data in this study are available from the corresponding author upon request. The data are not publicly available due to privacy considerations.

## Abbreviations

The following abbreviations are used in this manuscript:

AOI	Areas of Interest
FDR	False Discovery Rate
GLMM	Generalized Linear Mixed-Effects Model
HT	Human Translation
L1	First Language
L2	Second Language
LL	Log-Likelihood
LM	Language Model
LLM	Large Language Model
LMM	Linear Mixed-Effects Model
NMT	Neural Machine Translation
PE	Post-Editing

## Appendix A. Predictor Definitions and Feature Glossary

**Table A1:** Surprisal predictors used in the analysis.

Symbol	Definition	Interpretation
$S_{\mathrm{lm}}\left(u\right)$	$-\log p_{\mathrm{lm}}\left(x_{i}|x_{<i}\right)$, aggregated over source unit *u*.	Source-side predictability cost under left-to-right monolingual context; higher values indicate greater expected processing demand.
$S_{\mathrm{lm}}\left(v\right)$	$-\log p_{\mathrm{lm}}\left(y_{j}|y_{<j}\right)$, aggregated over target unit *v*.	Target-side predictability cost under left-to-right monolingual context.
$S_{\mathrm{mt}}\left(v\right)$	$-\log p_{\mathrm{mt}}\left(y_{j}|x_{1:m},y_{<j}\right)$, aggregated over target unit *v*.	Translation surprisal under source-conditioned decoding; higher values indicate greater bilingual generation uncertainty.

Note. Definitions correspond to Equations (1)–(3). At the segment level, surprisal is the sum of token-wise surprisals inside each aligned segment.

**Table A2:** Encoder self-attention predictors.

Symbol	Definition	Interpretation
$f_{u,u}^{e}$	Encoder attention mass from source token *u* to itself, $\alpha_{u,u}^{e}$.	Local source focus on the current token.
$f_{u,\bar{u}}^{e}$	Encoder attention mass from source token *u* to other source positions, $\sum_{i\neq u}\alpha_{u,i}^{e}$.	Breadth of contextual integration across source tokens.
$f_{u,\mathrm{eos}}^{e}$	Encoder attention mass from source token *u* to source EOS, $\alpha_{u,\mathrm{eos}}^{e}$.	Tendency to allocate source attention to sequence boundary information.
$H_{u,x}^{e}$	Entropy of encoder attention from source token *u*, $-\sum_{i}{\tilde{\alpha }}_{u,i}^{e}\log{\tilde{\alpha }}_{u,i}^{e}$, where ${\tilde{\alpha }}_{u,i}^{e}$ renormalizes $\alpha_{u,i}^{e}$ over the source positions.	Diffuseness of source-side attention distribution.
$f_{\bar{u},u}^{e}$	Total incoming encoder attention to source token *u*, $\sum_{i\neq u}\alpha_{i,u}^{e}$.	Global salience of a source token within source encoding.

Note. $\alpha_{u,i}^{e}$ denotes encoder self-attention from source token *u* to source position *i*; eos is source EOS.

**Table A3:** Cross-attention predictors.

Symbol	Definition	Interpretation
$f_{y,u}^{c}$	Total cross-attention mass from the target sequence to source token *u*, $\sum_{v=1}^{n}\alpha_{v,u}^{c}$.	Strength of target-to-source alignment for a given source token.
$f_{v,\mathrm{eos}}^{c}$	Cross-attention mass from target token *v* to source EOS, $\alpha_{v,\mathrm{eos}}^{c}$.	Allocation of source-conditioned attention to sequence boundary at target step *v*.
$H_{v,x}^{c}$	Entropy of cross-attention at target step *v*, $-\sum_{i}{\tilde{\alpha }}_{v,i}^{c}\log{\tilde{\alpha }}_{v,i}^{c}$, where ${\tilde{\alpha }}_{v,i}^{c}$ renormalizes $\alpha_{v,i}^{c}$ over the source positions.	Sharpness vs. diffuseness of source alignment during target decoding.

Note. $\alpha_{v,i}^{c}$ denotes cross-attention from target token *v* to source position *i*.

**Table A4:** Decoder self-attention predictors.

Symbol	Definition	Interpretation
$f_{v,v}^{d}$	Decoder self-attention mass from target token *v* to itself, $\alpha_{v,v}^{d}$.	Local target focus at the current decoding step.
$f_{v,\bar{v}}^{d}$	Decoder self-attention mass from target token *v* to previous target positions, $\sum_{j<v}\alpha_{v,j}^{d}$.	Reliance on preceding target context.
$H_{v,\bar{v}}^{d}$	Entropy over previous-target decoder attention, $-\sum_{j<v}{\tilde{\alpha }}_{v,j}^{d}\log{\tilde{\alpha }}_{v,j}^{d}$, where ${\tilde{\alpha }}_{v,j}^{d}$ is renormalized over j<v.	Dispersion of contextual weighting over previously generated target tokens.

Note. $\alpha_{v,j}^{d}$ denotes decoder self-attention from target token *v* to target position *j*; the renormalization ${\tilde{\alpha }}_{v,j}^{d}$ for $H_{v,\bar{v}}^{d}$ is defined in Appendix A.3.

### Appendix A.1. Features Derived from Encoder Self-Attention

Let $\alpha_{u,i}^{e}$ denote encoder attention from source token *u* to source position *i*, and let eos be source EOS.

(A1) $$f_{u,u}^{e}=\alpha_{u,u}^{e}.$$

(A2) $$f_{u,\bar{u}}^{e}=\sum_{i\neq u}\alpha_{u,i}^{e}.$$

(A3) $$f_{u,\mathrm{eos}}^{e}=\alpha_{u,\mathrm{eos}}^{e}.$$

(A4) $$H_{u,x}^{e}=-\sum_{i}{\tilde{\alpha }}_{u,i}^{e}\log{\tilde{\alpha }}_{u,i}^{e},\;{\tilde{\alpha }}_{u,i}^{e}=\frac{\alpha_{u,i}^{e}}{\sum_{i^{\prime }}\alpha_{u,i^{\prime }}^{e}}.$$

(A5) $$f_{\bar{u},u}^{e}=\sum_{i\neq u}\alpha_{i,u}^{e}.$$

### Appendix A.2. Features Derived from Cross-Attention

Let $\alpha_{v,i}^{c}$ denote cross-attention from target token *v* to source position *i*; the subscript *y* indicates summation over the full target sequence $y_{1:n}$.

(A6) $$f_{y,u}^{c}=\sum_{v=1}^{n}\alpha_{v,u}^{c}.$$

(A7) $$f_{v,\mathrm{eos}}^{c}=\alpha_{v,\mathrm{eos}}^{c}.$$

(A8) $$H_{v,x}^{c}=-\sum_{i}{\tilde{\alpha }}_{v,i}^{c}\log{\tilde{\alpha }}_{v,i}^{c},\;{\tilde{\alpha }}_{v,i}^{c}=\frac{\alpha_{v,i}^{c}}{\sum_{i^{\prime }}\alpha_{v,i^{\prime }}^{c}}.$$

### Appendix A.3. Features Derived from Decoder Self-Attention

Let $\alpha_{v,j}^{d}$ denote decoder self-attention from target token *v* to target position *j*.

(A9) $$f_{v,v}^{d}=\alpha_{v,v}^{d}.$$

(A10) $$f_{v,\bar{v}}^{d}=\sum_{j<v}\alpha_{v,j}^{d}.$$

(A11) $$H_{v,\bar{v}}^{d}=-\sum_{j<v}{\tilde{\alpha }}_{v,j}^{d}\log{\tilde{\alpha }}_{v,j}^{d},\;{\tilde{\alpha }}_{v,j}^{d}=\frac{\alpha_{v,j}^{d}}{\sum_{j^{\prime }<v}\alpha_{v,j^{\prime }}^{d}}.$$

## Appendix B. Random-Effects Structure Selection

Every comparison began from the correlated maximal structure and was simplified only when the model failed to converge or was singular. Table A5 reports the structure ultimately fitted in each model family. Across the 666 main-effect and residual attention comparisons, 341 (51%) retained a maximal structure (256 correlated, 85 uncorrelated), 78 used by-participant slopes, and 247 reached the random-intercept floor. The floor was reached almost entirely by the sparse duration and post-editing subsets, where slope variances could not be estimated. Of these 247 comparisons, 81 had a near-zero (boundary) variance estimate; their fixed-effect estimates were stable, and the corresponding effects were interpreted cautiously.

**Table A5:** Final random-effects structure across model families, after the maximal-to-reduced recovery ladder. Counts are numbers of base/augmented comparisons.

Family	Corr. Max.	Uncorr. Max.	Part. Slope	RI
Main effects	134	47	48	149
Residual attention	122	38	30	98
Interaction ^a^	58	8	n/a	18
Two-part duration ^b^	33	38	50	131

Note. Corr. max. = correlated maximal; Uncorr. max. = uncorrelated maximal; Part. slope = by-participant slope; RI = random intercepts. ^a^ Interaction models carry by-participant slopes only because sentence items are nested within task and direction; the correlated and uncorrelated columns therefore denote by-participant slopes, and the separate by-participant slope reduction step does not apply (n/a). ^b^ Two-part counts pool the logistic edit occurrence and Gaussian positive duration parts across all group subsets.

## Appendix C. Task Materials

This appendix provides the source texts used in all four task conditions and the drafts supplied in the post-editing conditions.

### Appendix C.1. Source Texts

**Text T1 (English)**

The problem is, while several tools seem to be gaining ground in computer models, laboratories, and even real-world experiments, public discussion has not kept pace with their advancement. To date, there has been too little transparency and international dialog around the progress, feasibility, risks, and benefits of these efforts. Climate engineering and current mitigation and adaptation efforts are not mutually exclusive. Experts generally agree that these new technological approaches alone are unlikely to provide adequate protection from the dangers posed by rising global temperatures.

**Text T2 (Chinese)**

**Text P1 (English)**

Design, another IP right, enables teams, organizers of sporting competitions and sports brands to develop and promote their unique and distinct identity and for fans to distinguish between them. And trademarks, which underpin sports branding, are an exceptionally important IP right for teams and athletes to differentiate themselves and stand apart in a highly competitive market. Trademark rights are critical in allowing individuals players and teams to gain a monetary reward from, for example, merchandising-including apparel, accessories, footwear and more-and sponsorship deals.

**Text P2 (Chinese)**

### Appendix C.2. Drafts for Post-Editing

**Draft P1 (Chinese)**

**Draft P2 (English)**

In China, the consequences of land desertification are profound: it intensifies the conflict between humans and land, reduces living space for humans, and causes a continuous decline in arable land. This exacerbates the growing imbalance between human needs and the land’s capacity to support them. Furthermore, it increases the frequency and severity of natural disasters, worsens the ecological environment, and threatens human survival conditions. Desertification is one of the primary causes of the recent surge in sandstorms across China, the rapid loss of biodiversity in desertified regions, and the frequent drought and flood disasters along the middle and lower reaches of the Yellow River. In desertified areas, vegetation is drastically reduced, and many species are either endangered or on the verge of extinction.
